# Assessing population structure and migration patterns of wild boar (*Sus scrofa*) in Japan

**DOI:** 10.1038/s41598-023-48215-0

**Published:** 2023-12-01

**Authors:** Kotaro Sawai, Aisaku Arakawa, Masaaki Taniguchi, Bo Xiao, Miwa Sawai, Makoto Osaki, Emi Yamaguchi, Yoko Hayama, Yoshinori Murato, Yumiko Shimizu, Sonoko Kondo, Takehisa Yamamoto

**Affiliations:** 1 Epidemiology and Arbovirus Group, Division of Transboundary Animal Disease Research, National Institute of Animal Health, National Agriculture and Food Research Organization, 3-1-5 Kannondai, Tsukuba, Ibaraki 305-0856 Japan; 2 Meat Animal Biosystems Group, Division of Meat Animal and Poultry Research, Institute of Livestock and Grassland Science, National Agriculture and Food Research Organization, 2 Ikenodai, Tsukuba, Ibaraki 305-0901 Japan; 3 Virus Group, Division of Infectious Animal Disease Research, National Institute of Animal Health, National Agriculture and Food Research Organization, 3-1-5 Kannondai, Tsukuba, Ibaraki 305-0856 Japan; 4 Division of Hygiene Management Research, National Institute of Animal Health, National Agriculture and Food Research Organization, 3-1-5 Kannondai, Tsukuba, Ibaraki 305-0856 Japan

**Keywords:** Population genetics, Genome informatics

## Abstract

Geographical wildlife patterns reflect historical range expansion and connectivity and provide insights into wildlife population management. In our large-scale phylogeographic population analysis of wild boars (*Sus scrofa leucomystax*) in Japan, we identified 15 clusters using 29 microsatellite markers, each structured within a range of approximately 200 km. This suggests that evolution was essentially driven by isolation by distance, and that the range of gene flow was limited. One cluster contained subpopulations located approximately 900 km apart, indicating the occurrence of past anthropogenic introductions. Moreover, we estimated effective migration to visualize the geographic genetic population diversity. This analysis identified six potential barriers, one of which involved large plains and mountainous areas in the Kanto region of eastern Japan. This barrier likely persisted in the two eastern clusters for an extended period, restricting migration to the neighboring areas. Overall, our study sheds light on the demographic history of wild boar in Japan, provides evidence of past anthropogenic introductions from distant areas, and highlights the importance of geographic barriers in shaping genetic diversity and population dynamics. This knowledge will be beneficial for forming informed wildlife management strategies toward the conservation of genetic integrity and ecological balance of wild boar populations in Japan.

## Introduction

The wild boar (*Sus scrofa*) is one of the most widely distributed terrestrial mammal species worldwide. These boars originated from islands in Southeast Asia and colonized various areas of Eurasia during several migration waves until the early Holocene^1,2^. Wild boars are classified into 16 subspecies based on their morphological characteristics and are found in Asia, Europe, and North Africa^3^; two subspecies exist in Japan, the Japanese wild boar (*Sus scrofa leucomystax*) and Ryukyu wild boar (*Sus scrofa riukiuanus*). These two subspecies are genetically distant and inhabit separate areas of Japan^4^. An ancestor of the Japanese wild boar spread from Southeast to Northeast Asia during the mid-to-late Pleistocene^5^ and expanded its habitats in Japan. In contrast, the Ryukyu boar migrated from Taiwan and elsewhere, unlike the Japanese boar, which migrated across the continent^6^.

Even when population densities are temporarily reduced by infectious disease or hunting, number of this species can increase rapidly owing to its high reproductive potential. In fact, over the past four decades, the Japanese wild boar population has progressively expanded by approximately 1.9 times^7^, resulting in substantial damage to food crops and approximately USD 34 million worth of damage in 2021^8^. Notably, the wild boar population density of Japan is higher than that of other countries^9^. For the proper management of wild boars, an understanding of their present geographical distribution pattern is crucial.

Genetic characterization has been widely used to determine the population structure and patterns of gene flow in wildlife. DNA markers, such as mitochondrial DNA (mtDNA), microsatellites (simple sequence repeats [SSRs]), and single nucleotide polymorphisms (SNPs) using whole-genome sequences, are commonly used to analyze the population structure^10,11^. In comparison to other genetic analysis methods, mtDNA analysis is generally considered easier in terms of sample collection and laboratory procedures^12^. Additionally, mtDNA analysis provides only maternal genetic information, and inferences are based on a single short haploid locus. In contrast, because microsatellite loci from SSRs carry highly polymorphic, neutral, and biparental gene flow, they can provide relatively finer patterns than mtDNA for detecting recent population differences and admixtures^13,14^. However, population studies using SSR markers or SNPs are expensive and time-consuming.

Numerous extensive phylogeographic investigations of wild boars have been conducted in Japan, primarily employing the mtDNA control region^15,16^. However, the latest comprehensive study by Watanobe et al., which covered eastern Japan, dates back to 2003. Furthermore, as previously mentioned, the rapid expansion of the wild boar population’s range in Japan over the last four decades^7^ may have led to alterations or increased complexity in the population structure compared to the period of these earlier studies.

**Figure 1:**
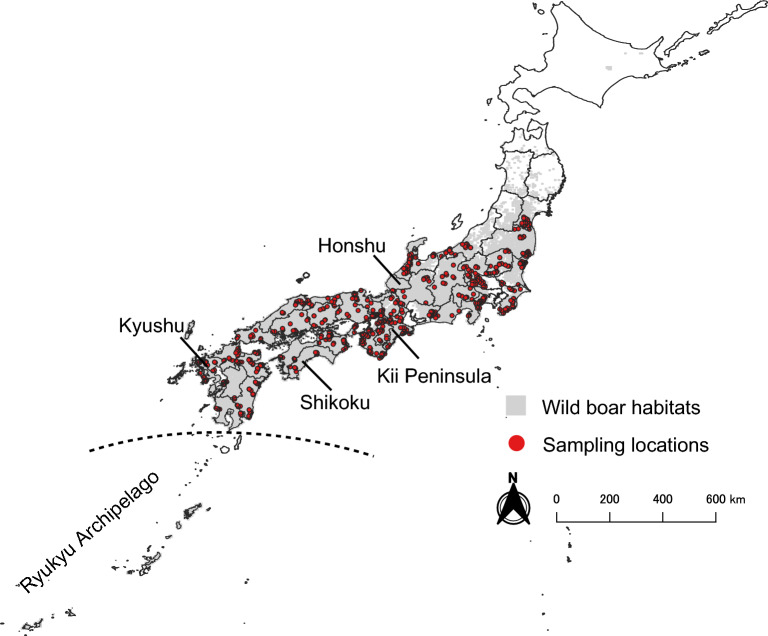
Geographical distribution of 1062 wild boar samples collected in Japan from 2014 to 2020. Red points represent the locations where the wild boar samples were collected. Grey areas represent wild boar habitats in Japan as surveyed by the Japanese Ministry of Environment^17^. The details of each site are listed in Supplementary Data 3. The dashed line indicates the boundary between the habitats of Japanese wild boars (*Sus scrofa leucomystax*) and Ryukyu wild boars (*Sus scrofa riukiuanus*). Geographical data regarding the administrative boundaries were downloaded from the National Land Numerical Information download service, which is provided by the Ministry of Land, Infrastructure, Transport, and Tourism of Japan^18^. The map was created using QGIS version 3.28.10^19^.

In this study, we conducted large-scale sampling covering Japanese wild boar habitats, comprising a total of 1062 individuals, to elucidate regional patterns of genetic population structure using 30 microsatellite loci (Fig. 1). We report the population structure and migration patterns of wild boar in Japan. Our study provides insights into the historical processes of biological range expansion, connectivity and dispersal barriers in wild boar and will be useful for wild boar management in Japan. Therefore, this study will be helpful as an invaluable reference data for future investigations of the genetic structure of the wild boar population on a large scale during and after the process of its recovery.

## Results

### Microsatellite genotyping

Except for S0090, 29 loci in > 99% of individuals were genotyped. MICROCHECKER identified no loci with null alleles common to all populations. We identified no paired loci with a linkage disequilibrium ($\overline{r}$D) greater than 0.2, suggesting low covariation among the loci (Supplementary Data 1). Consequently, we used 29 locus alleles (excluding S0090) in subsequent analyses. All loci showed high levels of polymorphism, resulting in a total of 471 alleles, ranging from 7 (Sw122) to 35 (S0097), that were detected for these 29 loci; we detected a mean of 16 alleles per locus. The mean observed heterozygosity (H_O_) and expected heterozygosity (H_E_) ranged from 0.16 to 0.78 and 0.22 to 0.92, respectively (Supplementary Data 2). All loci deviated significantly from the Hardy–Weinberg equilibrium (HWE; *p* < 0.05).

### Population structure

**Figure 2:**
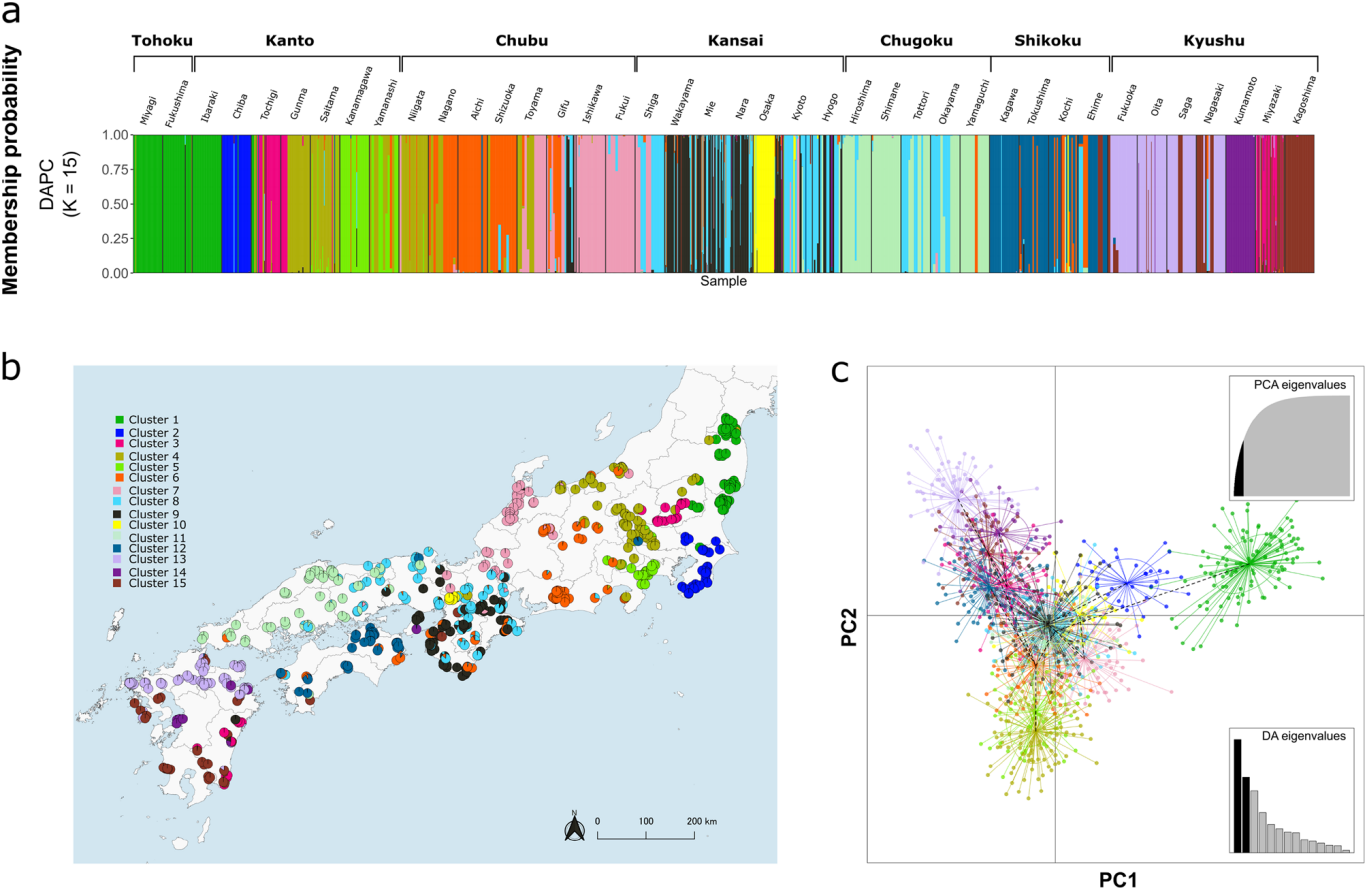
Population structure of *Sus scrofa leucomystax* in Japan. (**a**) Bar plot of individual ancestry proportions for the genetic clusters inferred using discriminant analysis of principal components (DAPC). Estimated Bayesian genetic structures were based on 29 microsatellite loci with K = 15. (**b**) Pie charts represent the proportion of the assignment to each cluster based on the DAPC results shown in (**a**). Geographical data regarding the administrative boundaries were downloaded from the National Land Numerical Information download service, which is provided by the Ministry of Land, Infrastructure, Transport, and Tourism of Japan^18^. This map was created using QGIS version 3.28.10^19^. (**c**) Scatterplot of DAPC with principal components, PC1 and PC2.

Discriminant analysis of principal components (DAPC) indicated that K = 15 was the most appropriate number of clusters for describing the dataset with the lowest Bayesian information criterion values (Supplementary Fig. 1a). Forty-two principal components (PCs) were retained via cross validation (Supplementary Fig. 1b). The genetic structure pattern showed that individuals close to each other were essentially in the same cluster; 90% clustering occurred within approximately 200 km (median 146 km) (Fig. 2a,b, Supplementary Fig. 2). The first discriminant function (PC1) separated Clusters 1 and 2 from the rest, whereas the second discriminant function (PC2) separated the clusters according to the longitude of the locations from where the individuals were collected (Fig. 2c). Clusters 6, 8, 9, 10, and 11 overlapped geographically and genetically, whereas the remaining clusters were geographically exclusive of the surrounding clusters. Notably, the geographical distance between individuals belonging to Cluster 3 showed a peak at 100 and 900 km, respectively, whereas the other clusters showed a single peak (Supplementary Fig. 2). In Cluster 3, one spatial cluster was located in the Kanto region, whereas the other was found in the Kyushu region, a large western island separated by the sea (Fig. 2a,b).

### Spatial autocorrelation

**Figure 3:**
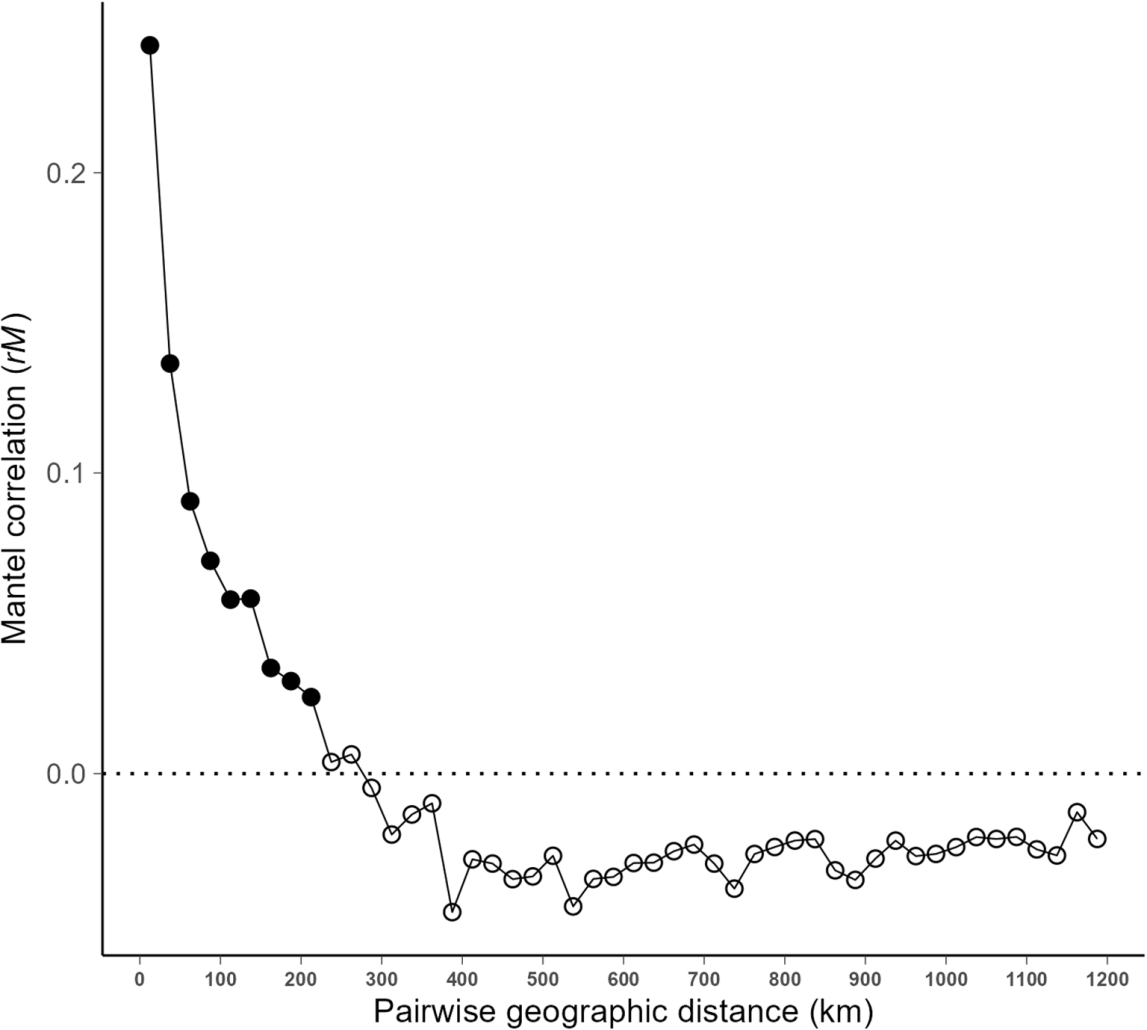
Mantel correlogram depicting the association between genetic and geographic distances among pairs of individuals within each distance class of 20 km. Black solid points indicate significant association, whereas white empty points indicate no significance or a significantly negative correlation value (*p* < 0.05).

We observed a decrease in spatial autocorrelation as geographical distance increased. Moreover, we observed a significant positive correlation for spatial distances of up to 200 km (*p* < 0.05). When the distance between individuals was greater than 200 km, they were no longer related (i.e., spatially independent) (Fig. 3).

### Potential geographical barrier

**Figure 4:**
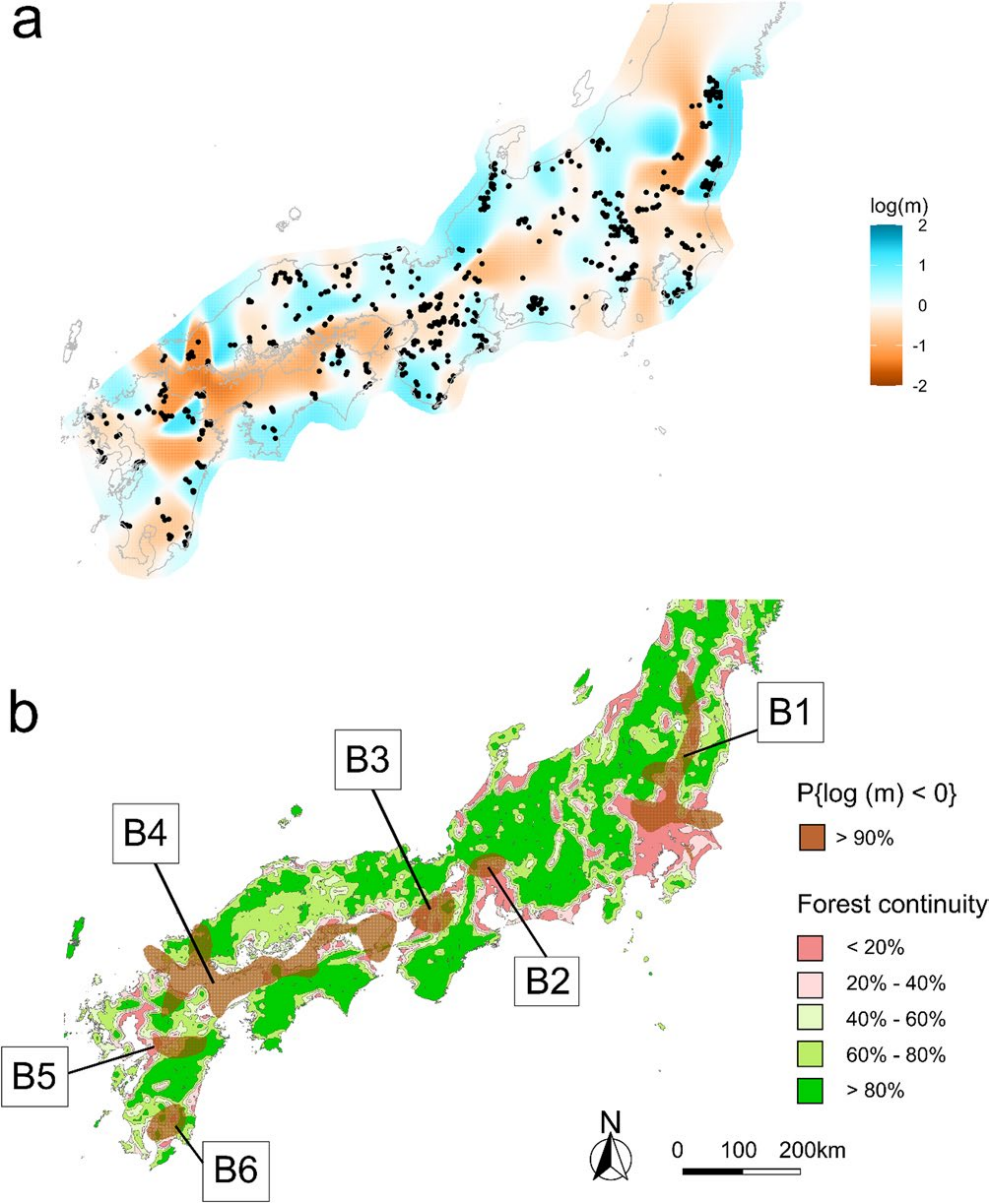
Effective migration surface distribution and comparison with forest continuity for identification of geographical barriers. Geographical data regarding the administrative boundaries were downloaded from the National Land Numerical Information download service, which is provided by the Ministry of Land, Infrastructure, Transport, and Tourism of Japan^18^. (**a**) Blue regions indicate a higher effective migration rate, whereas orange regions indicate lower effective migration rate than expected. (**b**) Forest continuity map^20^ overlaid with the geographical barriers that have > 90% probability shown as B1–6. The maps were created using R 4.0.2.

Because the estimated effective migration surface (EEMS) analysis method visualizes migration patterns and tests spatial genetic structures using genomic data and surrounding locations defined by the density of demes, we used 1000 demes, the maximum number allowed by the program. As all five Markov chain Monte Carlo runs of the program converged (Supplementary Fig. 3), we considered this analysis reliable. Our EEMS analysis showed potential barriers to migration not only in the sea separating Honshu from Shikoku and Kyushu, but also in terrestrial areas (Fig. 4a). Of these potential barriers, we identified six (labelled B1–B6) with greater than 90% posterior probability in the Bayesian estimation of migration parameters (Fig. 4b). We observed that six barriers were aligned with forest discontinuity areas (B1–3, B5–6) or topographical obstacles, such as the sea (B4). We identified three potential geographical barriers (B1–3) in Eastern Honshu. B1 was located in mountainous areas and the large southern plains. B2–3 was located in the plains surrounding urban areas, such as Nagoya and Osaka. In the Kyushu region, there were two areas with low migration rates (B5–6) and low forest connectivity. In contrast, we found no clear or significant geographical barriers in the Shikoku region or western Honshu.

## Discussion

In this study, we gathered samples from 1062 wild boars inhabiting various regions across Japan where wild boar populations are found. We used 29 microsatellite loci to reveal the genetic population structure at high resolution and identify the potential geographic barriers preventing the migration of wild boars. We observed a significant deviation from the HWE for all loci, which is consistent with the findings of other studies^21,22^, as there may be greater differences between subpopulations in large areas. From our multivariate clustering (DAPC) and EEMS analyses, we identified 15 genetically meaningful clusters; individuals belonging to each cluster were closely distributed, except for Cluster 3. Additionally, the clusters were separated by geographical features such as forest discontinuity, plains, and sea.

Cluster 1 individuals were exclusively located in an area separated from other areas by barrier B1 at its western and southern borders (Figs. 2 and 4). Murase et al.^23^ also reported that the wild boar populations in this area were a single population. B1 was identified as a continuous gap in mountainous areas, stretching in the northern and eastern directions (Fig. 4). Our findings are also consistent with those of a previous study conducted at the local scale, which suggested that rivers and urbanized areas act as natural migration barriers^24^. However, as several land uses are included in B1, it is unclear which land use category hinders migration the most.

In the area south of B1, individuals belonging to Cluster 2 were found (Figs. 2 and 4). This cluster was located on a peninsula with a large plain in the north and the surrounding sea, indicating that it was also an isolated population. However, in contrast to Cluster 1, the population in this peninsula became extinct in the 1970s due to indiscriminate hunting and infectious diseases, such as classical swine fever (CSF)^23^. Subsequently, in the mid-1980s, wild boars were anthropogenically reintroduced into the southern region of the peninsula for hunting purposes, and these wild boars subsequently expanded their habitat into the northern area^23^. Because EEMS is not suitable for examining the ancestral relationship between subpopulations, further studies focusing on SNPs are required to identify the genetic source of Cluster 2.

Although EEMS analysis identified the existence of B2 and B3 in the north of the Kii Peninsula, individuals in Clusters 6, 8, and 9 were located on both sides of B2 and B3 (Figs. 2 and 4; see also Fig. 1, which includes the peninsula names). Thus, these barriers are not completely responsible for the fragmentation of gene flow in different regions. Additionally, we did not identify any clear geographical barriers west of the Kansai region, suggesting that individuals belonging to Clusters 6–11 were more likely to migrate among each other than to the other clusters. Although Watanobe et al.^5^ reported the Japanese Alps in the center of Honshu as a potential geographic barrier to migration, we found no evidence of major discontinuities consistent with the Japanese Alps, which lie between B1 and B2. Our results indicate that gene flow is active among several subpopulations because of the high forest continuity in the areas surrounding B2 and B3. Furthermore, several studies have reported that landscapes such as forest cover or small-scale agriculture facilitate the dispersal and genetic homogenization of wild boars^25,26^.

B4 coincides with the sea between the Honshu, Shikoku, and Kyushu islands (Fig. 4; see also Fig. 1, which includes the names of these islands). Although mtDNA analysis has only identified the sea separating Honshu and Kyushu as a genetic boundary^5^, our analysis showed that this sea may also act as a barrier to dispersal. On Kyushu Island, B5 and B6 coincided with the plains with interrupted forest connectivity (Fig. 4), suggesting that they contributed to the population formation of Clusters 3, 13, 14, and 15 (Fig. 2).

Mantel analysis demonstrated a significant positive spatial autocorrelation between individuals (*p* < 0.05) located at distances of < 200 km (Fig. 3). This finding is consistent with the distance reported in other studies^26,27^. Furthermore, when plotting the distribution of the pairwise distances between individuals in each cluster, the 90th percentile for each cluster was < 200 km, except in Cluster 3 (Supplementary Fig. 2). These results indicate that wild boar subpopulations with genetic similarity were created with a diameter of approximately 200 km. Notably, most individuals in Cluster 3 were distributed in two distant locations (Supplementary Fig. 2): the Kanto (n = 32) and the Kyusyu (n = 33) regions, approximately 900 km away. Several studies have reported that the J12 haplotype, which was identified using mtDNA analysis in these studies, was also present in geographically distant areas, i.e., the Kanto, Kyusyu, and the Shikoku regions^5,23, 28^. The wild boar population in the western part of the Tochigi Prefecture in the Kanto region became extinct in approximately 1897 because of unregulated hunting and a CSF epidemic^28,29^. Subsequently, the population increased in southwestern Tochigi, either through range expansion from Gunma, which is located as western prefecture or translocation from distant areas mediated by humans^29,30^. Considering these circumstances, it is likely that the individuals belonging to Cluster 3 in the Tochigi Prefecture in this analysis were a natural expansion of the range after a previous human-mediated translocation from Miyazaki to the Gunma Prefecture. As discussed above for Cluster 3, further analysis focusing on SNPs is required to genetically prove this hypothesis.

However, caution should be exercised when interpreting the results of this study. First, even though we performed microsatellite analysis of a sufficient number of loci to identify their genetic clusters and elucidate geographical barriers to the distribution of wild boar populations, using SNPs or whole genome analysis could provide finer-scale population structures and evolutionary relationships among subpopulations than SSRs. Furthermore, the representation of genetic variation representation could have been biased based on the use of a reference SSR panel in a large-scale study. In addition, “size homoplasy”, specifically individuals with the same allele size but different ancestries, might have occurred in the microsatellite analysis. In fact, this phenomenon is quite common owing to the high mutation rate and the genotyping performed using fragment lengths that contain repetitive regions and flanking regions^31,32^. Thus, for each marker, not all allele value differences corresponded to repeat units. Therefore, further investigations are required to confirm our results, such as considering the chronological aspects between strains. Second, although several studies have reported the influence of topographical obstacles, such as rivers and freeways, on wild boar populations^24,33^, we did not explicitly consider these parameters here. Moreover, the geographical resolution used for analyzing the geographical barriers in this study seemed to be insufficient (1000 demes; each deme had an edge of approximately 15 km) to evaluate the influence of specific topographical obstacles. Further studies with more samples are required to increase the analysis resolution and identify the specific obstacles acting as barriers to wild boar migration.

In summary, our study demonstrates the population structure and migration patterns of wild boar in Japan. Generally, wild boar management in Japan is mainly conducted at the prefectural level, and their migration or population structure does not correspond to a specific administrative unit. Moreover, it is feasible for wild boar and domestic pigs to interbreed. In fact, the presence of hybrids has been reported in Japan^4,34, 35^. Thus, when farms have inadequate biosecurity, there is a risk that infectious diseases might also be easily transmitted. This underscores the necessity for cooperation beyond the municipal level for proper management. From the viewpoint of infectious disease control, the genetic boundaries identified in this study will be useful for planning or evaluating strategies to control the spread of infectious diseases particularly when inter-individual transmission is considered the main route of infection (i.e. in the early stages of an epidemic)^36–38^. In 2018, a CSF outbreak occurred in Japan and is still spreading, not only in domestic pigs but also in wild boars. Mitigating wild boar infection will be key to controlling CSF spread through the use of an oral vaccine or wild boar depopulation^39^. Additionally, the threat of the African swine fever (ASF) virus entering Japan is increasing, owing to the recent outbreak of ASF across East Asian countries. Hence, the results of the present analysis could provide valuable insights for considering measures to control the subsequent spread of infection, such as a possible ASF outbreak. Moreover, since this study was performed prior to the CSF outbreak, further validation, based on assessing changes in the population structure pre- and post-CSF outbreak, would also provide valuable information regarding the mechanisms of disease spread.

## Methods

### Ethical approval

All wild boar sampling activities were conducted from hunted and dead wild boars as official surveillance activities by licensed hunters and the Prefectural Animal Hygiene Service Center located in each prefecture. Therefore, no sampling activity from living animals was included in this study and no ethical approval was required.

### Study area and sampling collection

We analyzed 1062 blood or tissue samples (ear and tongue) from hunted and dead wild boars collected by licensed hunters throughout the habitat areas identified in the survey between 2014 and 2020^7^ (Fig. 1, Supplementary Fig. 4 and Supplementary Data 3). We recorded the date, location, estimated age, weight, and hunting method associated with each animal. Juveniles caught simultaneously were excluded to ensure sample independency. Blood samples were sent to the National Institute of Animal Health, NARO, where they were maintained at 4 °C and centrifuged for serum extraction. Until processing, tongue and serum samples were stored at -20 °C, and ear samples were stored in alcohol. The extraction of genomic DNA from the samples was done using the Qiagen DNeasy Blood and Tissue Kit (Qiagen, Hilden, Germany) according to the manufacturer's instructions. We then performed multiplex PCR amplification using 30 microsatellite markers (Supplementary Data 4), as recommended by the ISAG/FAO^40^, using AmpliTaq Gold (Applied Biosystems, Waltham, MA, USA). The PCR mixture included 10 ng genomic DNA template, 1 × final concentration of PCR buffer, 200 µM final concentration dNTP mix, 0.1 µM each of forward and reverse primers, and 1 unit Taq DNA polymerase in a final reaction volume of 20 µl. Thermal cycling parameters for multiplex PCR were set with initial denaturation at 94 °C for 9 min, followed by 40 cycles at 94 °C for 30 s, annealing at 55 °C for 30 s, and extension at 72 °C for 1 min. After the PCR, two to three multiple PCRs, ensuring that the amplicon sizes and dyes did not overlap, were mixed and diluted in 1/10 ratio, and then 1 µl of the multiplexed PCR mixture was dissolved in 9 µl HiDi-formamide (Applied Biosystems, Waltham, MA, USA). Typing was performed using capillary electrophoresis on an ABI 3130XL Genetic Analyser (Applied Biosystems, Waltham, MA, USA) with ROX400 as the internal size standard, and the allele size was determined using GeneMapper v4.0 (Applied Biosystems, Waltham, MA, USA).

### DNA extraction and genotyping

As minimizing missing information yields the most reliable results^41^, we excluded loci that could not be genotyped in > 1% of all samples. Potential errors of alleles were checked using the software MICROCHECKER v2.2.3^42^, applying the Brookfield 1 index^43^, and retained loci with values < 0.2^44,45^. We explored the linkage disequilibrium using the poppr package in R^46^, and excluded loci with a standard index of association ($\overline{r}$D) > 0.2. Based on the final dataset, the number of alleles, observed heterozygosity (H_O_), expected heterozygosity (H_E_), inbreeding coefficient (F_IS_), and deviation from the HWE were computed using the pegas and heirfstat packages^47,48^.

### Population structure

We analyzed the population structure using DAPC implemented in the adegenet package of R^49^. Compared with other widely used clustering programs, such as STRUCTURE, which relies on an explicit population genetic model^50^, the DAPC method is a model-free method, particularly for determining deviation from the HWE in genetic clustering analysis. Additionally, the DAPC method is more robust and suitable for assessment when the genetic structure is more or less clinal and under complex dispersal scenarios, such as those represented by stepping-stone and hierarchical stepping-stone models^49^. The optimal number of retained PCs were determined using cross validation methods to avoid overfitting of the data and creating a artificially large separation between clusters. We identified the optimal number of genetic clusters (K) that minimizes within-cluster variance using k-means estimation, as determined using the Bayesian information criterion, and estimated the probabilities of membership to each cluster for each sample. To assess the spatial aggregation of the samples, we calculated the geographical distance between pairs of individuals within each defined cluster.

### Spatial autocorrelation

Generally, genetic and geographic distances are correlated. To evaluate the relationship between genetic and geographic distances across space, we assessed the extent of spatial clustering in the samples using the vegan package in R. We calculated shared allele proportions (Dps) and geographic distances between pairs of individuals using distHaversine. We estimated correlograms of *r*-values for each class bin at a fixed distance of 20 km. We generated *p*-values using the permutational Mantel test (1000 permutations) and adjusted them using the Bonferroni procedure to correct the significance level for multiple comparisons.

### Geographical barriers

To detect potential geographic barriers and gene flow patterns, we used the EEMS analysis method, which assesses the bias in genetic structure due to isolation by distance and then produces a visual representation of effective migration rates^51^. This method does not require the use of environmental variables and can therefore be evaluated independently of the hypothesis-driven resistance surface approach. In turn, a map of the inferred effective migration patterns can be used to visualize the spatial genetic structure of large, complex samples. We set the number of demes to 200, 500, and 1000, and ran five independent analyses with 1 × 10^6^ burn-in Markov chain Monte Carlo steps and 1 × 10^7^ iterations. We combined the results of the five runs and plotted them using the rEEMSplots2 package in R (https://github.com/dipetkov/reemsplots2). We superimposed the areas estimated as barriers with a probability of > 90% on the forest continuity polygon to assess the relationship between the natural habitat of wild boar and geographical barriers^20^.

### Statistics and reproducibility

All statistical analyses are performed using either software packages as described or in R 4.0.2. All differences were considered statistically significant when *p* < 0.05 (see “Results” for details).

### Supplementary Information

Supplementary Figures.

Supplementary Information.

## Data Availability

The genotyping data generated in the present study are fully available at 10.6084/m9.figshare.24431806.

## Code availability

The original R codes for the analysis are available from the corresponding author upon request.

## References

[1] Larson G (2005). Worldwide phylogeography of wild boar reveals multiple centers of pig domestication. Science.

[2] Larson G (2010). Patterns of East Asian pig domestication, migration, and turnover revealed by modern and ancient DNA. Proc. Natl. Acad. Sci..

[3] Keuling O (2017). Ecology, Conservation and Management of Wild Pigs and Peccaries.

[4] Okumura N (2001). Genetic relationship amongst the major non-coding regions of mitochondrial DNAs in wild boars and several breeds of domesticated pigs. Anim. Genet..

[5] Watanobe T, Ishiguro N, Nakano M (2003). Phylogeography and population structure of the Japanese wild boar *Sus scrofa leucomystax*: Mitochondrial DNA Variation. Zool. Sci..

[6] Yoshikawa S (2016). Historical relationships among wild boar populations of the Ryukyu archipelago and other Eurasian regions, as inferred from mitochondrial cytochrome b gene sequences. Zool. Sci..

[7] Ministry of the Environment. Report on the national surveillance on the deer and wild boar habitats. http://www.env.go.jp/press/files/jp/115729.pdf (Ministry of the Environment, 2021).

[8] MAFF. Changes in crop damage by wildlife. https://www.maff.go.jp/j/seisan/tyozyu/higai/hogai_zyoukyou/ (Ministry of Agriculture, Forestry and Fisheries, 2020).

[9] Lewis JS (2017). Biotic and abiotic factors predicting the global distribution and population density of an invasive large mammal. Sci. Rep..

[10] Iacolina L (2018). Hotspots of recent hybridization between pigs and wild boars in Europe. Sci. Rep..

[11] Tong X (2020). Whole genome sequence analysis reveals genetic structure and X-chromosome haplotype structure in indigenous Chinese pigs. Sci. Rep..

[12] Brown WM, George M, Wilson AC (1979). Rapid evolution of animal mitochondrial DNA. Proc. Natl. Acad. Sci..

[13] Scandura M (2008). Ancient vs. recent processes as factors shaping the genetic variation of the European wild boar: Are the effects of the last glaciation still detectable?. Mol. Ecol..

[14] Choi SK (2014). Genetic structure of wild boar (*Sus scrofa*) populations from East Asia based on microsatellite loci analyses. BMC Genet..

[15] Watanobe T (1999). Genetic relationship and distribution of the Japanese wild boar (*Sus scrofa leucomystax*) and Ryukyu wild boar (*Sus scrofa riukiuanus*) analysed by mitochondrial DNA. Mol. Ecol..

[16] Watanobe T (2001). Ancient mitochondrial DNA reveals the origin of *Sus scrofa* from Rebun Island, Japan. J. Mol. Evol..

[17] Ministry of the Environment. Natural Environment Survey Web-GIS. http://gis.biodic.go.jp/webgis/

[18] Ministry of Land Infrastructure Transport and Tourism. National Land Numerical Information (NLNI). https://nlftp.mlit.go.jp/ksj/index.html (2018).

[19] QGIS Development Team. QGIS 3.28.10 Geographic information system. Open source geospatial foundation project. https://qgis.org/ja/site/ (2022).

[20] Ministry of the Environment. Continuous forest areas. https://www.biodic.go.jp/biodiversity/activity/policy/map/map03/index.html

[21] Scandura M, Iacolina L, Cossu A, Apollonio M (2011). Effects of human perturbation on the genetic make-up of an island population: The case of the Sardinian wild boar. Heredity (Edinb).

[22] Quinn CB (2022). Contrasting genetic trajectories of endangered and expanding red fox populations in the western U.S. Heredity (Edinb).

[23] Murase K (2015). Integrating analyses of population genetics and space-time information for wildlife management: An empirical study on Japanese wild boar populations. Mammal Study.

[24] Saito R (2022). Genetic population structure of wild boars (*Sus scrofa*) in Fukushima prefecture. Animals.

[25] Rutten A (2019). Analysing the recolonisation of a highly fragmented landscape by wild boar using a landscape genetic approach. Wildl. Biol..

[26] Reiner G, Rumpel M, Zimmer K, Willems H (2021). Genetic differentiation of wild boar populations in a region endangered by African swine fever. J. Wildl. Manag..

[27] McCann BE (2018). Molecular population structure for feral swine in the United States. J. Wildl. Manag..

[28] Ishiguro N, Nishimura M (2005). Genetic profile and serosurvey for virus infections of Japanese wild boars in Shikoku Island. J. Vet. Med. Sci..

[29] Nagata J (2006). Genetic characteristics of the wild boars in Tochigi prefecture and neighboring prefectures. Wildl. Tochigi Pref..

[30] Takahashi R (2018). Detection of inobuta from wild boar population in Japan by genetic analysis. Rev. Agric. Sci..

[31] Selkoe KA, Toonen RJ (2006). Microsatellites for ecologists: a practical guide to using and evaluating microsatellite markers. Ecol. Lett..

[32] Germain-Aubrey, C. C., Nelson, C., Soltis, D. E., Soltis, P. S & Gitzendanner, M. A. Are microsatellite fragment lengths useful for population-level studies? The case of Polygala lewtonii (Polygalaceae). *Appl. Plant Sci.* **4**(2). 10.3732/apps.1500115 (2016). 10.3732/apps.1500115 PMC4760751 26949579

[33] Tadano R, Nagai A, Moribe J (2016). Local-scale genetic structure in the Japanese wild boar (*Sus scrofa leucomystax*): Insights from autosomal microsatellites. Conserv. Genet..

[34] Naya Y, Horiuchi M, Ishiguro N, Shinagawa M (2003). Bacteriological and genetic assessment of game meat from Japanese wild boars. J. Agric. Food Chem..

[35] Anderson D (2021). Introgression dynamics from invasive pigs into wild boar following the March 2011 natural and anthropogenic disasters at Fukushima. Proc. R. Soc. B Biol. Sci..

[36] Conner MM, Miller MW (2004). Movement patterns and spatial epidemiology of a prion disease in mule deer population units. Ecol. Appl..

[37] Oyer AM, Mathews NE, Skuldt LH (2007). Long-distance movement of a white-tailed deer away from a chronic wasting disease area. J. Wildl. Manag..

[38] Lange M, Guberti V, Thulke H-H (2018). Understanding ASF spread and emergency control concepts in wild boar populations using individual-based modelling and spatio-temporal surveillance data. EFSA Support. Publ..

[39] Rossi S (2015). Controlling of CSFV in European wild boar using oral vaccination: A review. Front. Microbiol..

[40] Food and Agriculture Organization of the United Nations (FAO) (2011). Molecular Genetic Characterization of Animal Genetic Resources.

[41] Li Z, Gopal V, Li X, Davis JM, Casella G (2012). Simultaneous SNP identification in association studies with missing data. Ann. Appl. Stat..

[42] Van Oosterhout C, Hutchinson WF, Wills DPM, Shipley P (2004). micro-checker: Software for identifying and correcting genotyping errors in microsatellite data. Mol. Ecol. Notes.

[43] Brookfield JF (1996). A simple new method for estimating null allele frequency from heterozygote deficiency. Mol. Ecol..

[44] Dakin EE, Avise JC (2004). Microsatellite null alleles in parentage analysis. Heredity (Edinb).

[45] Chapuis M-P, Estoup A (2007). Microsatellite null alleles and estimation of population differentiation. Mol. Biol. Evol..

[46] Kamvar ZN, Tabima JF, Grünwald NJ (2014). Poppr: An R package for genetic analysis of populations with clonal, partially clonal, and/or sexual reproduction. PeerJ.

[47] Paradis E (2010). pegas: An R package for population genetics with an integrated-modular approach. Bioinformatics.

[48] Goudet J (2005). hierfstat, a package for R to compute and test hierarchical F-statistics. Mol. Ecol. Notes.

[49] Jombart T, Devillard S, Balloux F (2010). Discriminant analysis of principal components: A new method for the analysis of genetically structured populations. BMC Genet..

[50] Falush D, Stephens M, Pritchard JK (2003). Inference of population structure using multilocus genotype data: Linked loci and correlated allele frequencies. Genetics.

[51] Petkova D, Novembre J, Stephens M (2016). Visualizing spatial population structure with estimated effective migration surfaces. Nat. Genet..

