# Supplementary material for: Assessing population structure and migration patterns of wild boar (Sus scrofa) in Japan
Source: Sci Rep. 2023 Dec 1;13:21186. doi: 10.1038/s41598-023-48215-0 (PMC10692317; doi:10.1038/s41598-023-48215-0)
Supplement: Supplementary file 1 — Supplementary Figures. [file 41598_2023_48215_MOESM1_ESM.pdf]

Supplemental Information for:

Assessing population structure and migration patterns of wild boar (*Sus scrofa*) in Japan

Kotaro Sawai, Aisaku Arakawa, Masaaki Taniguchi, Bo Xiao, Miwa Sawai, Makoto Osaki, Emi Yamaguchi, Yoko Hayama, Yoshinori Murato, Yumiko Shimizu, Sonoko Kondo, Takehisa Yamamoto

Supplementary Figures:

Supplementary Figure 1 - Results of parameter using in discriminant analysis of principal components (DAPC).

Supplementary Figure 2 - Results of discriminant analysis of principal components (DAPC) analysis.

Supplementary Figure 3 - EEMS output.

Supplementary Figure 4 - Prefectures and regions of Japan used in this study.

Supplementary Data:

Supplementary Data 1 - Results of linkage disequilliberuim expressed as standardised index of association, measured with 999 resampling using the poppr package in R.

Supplementary Data 2 - Summary of locus characteristics of 29 microsatellites of wild boar in Japan.

Supplementary Data 3 - Allele values of wild boar collected between 2014 and 2020 in Japan.

Supplementary Data 4 - List of 30 microsatellite markers.

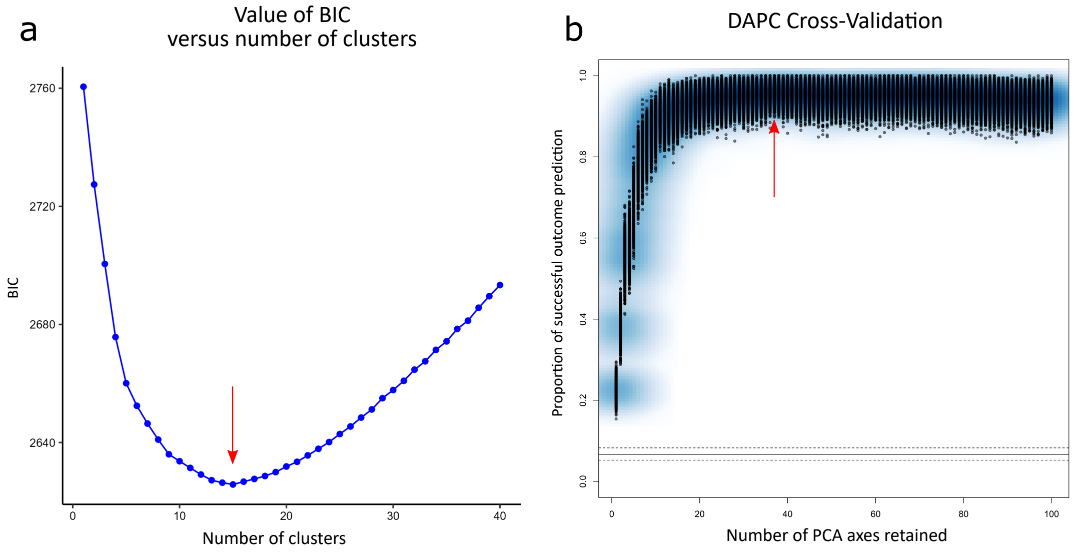

Supplementary Figure 1. Results of parameter using in discriminant analysis of principal components (DAPC).

(a) Baysian information criterion (BIC) values for each number of clusters. The lowest values were indicated by an arrow (K=15). (b) The plot of DAPC cross validation. The x-axis is the number of PCA axes retained for DAPC, and the y-axis is the proportion of successful outcome predictions. Individual replicates appear as point, and the density of these points in different regions of the plot is shown in blue.

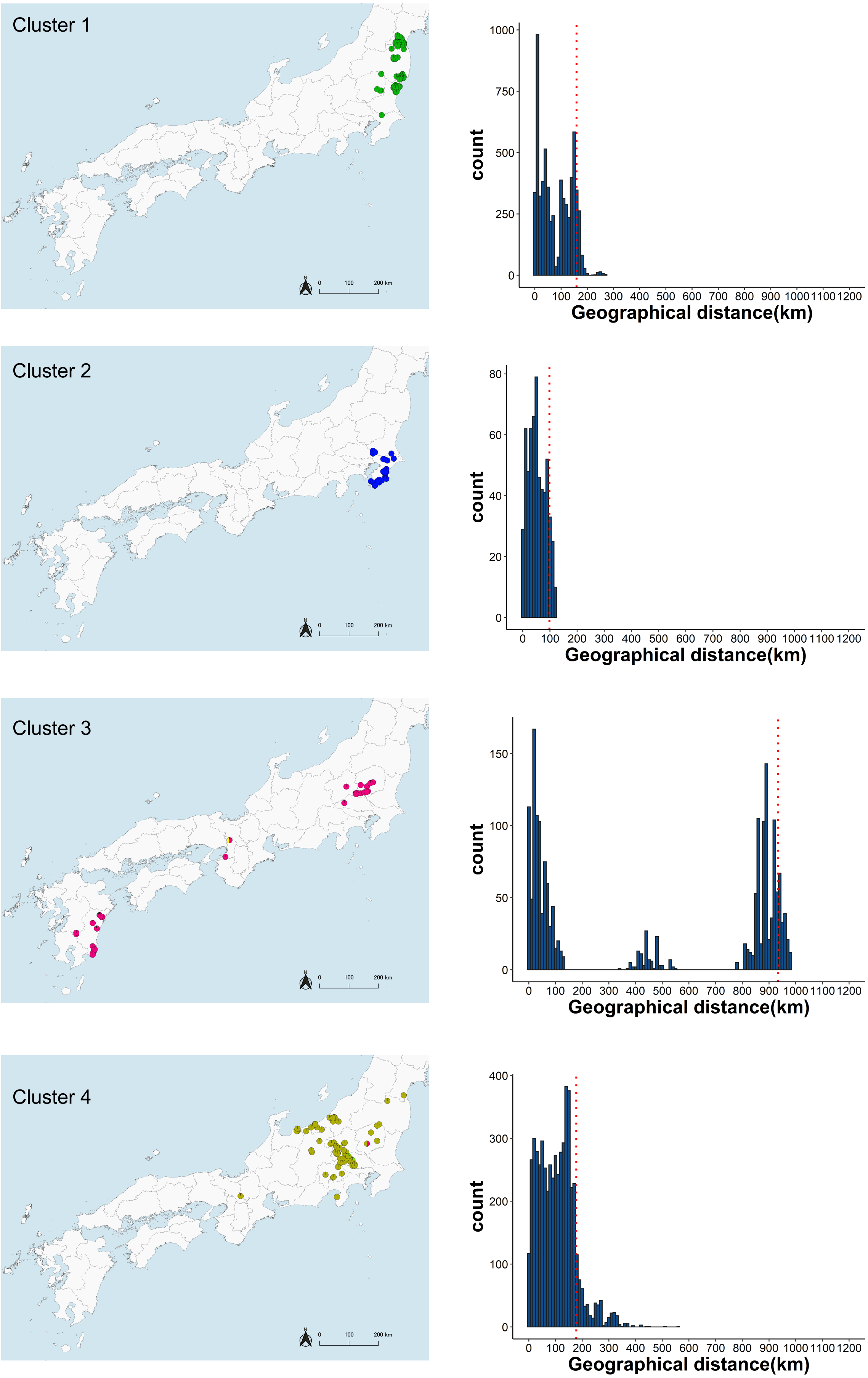

Cluster 1
0
100
200 km
1000
750
500
250
0
count
0 100 200 300 400 500 600 700 800 900 1000 1100 1200
Geographical distance(km)
Cluster 2
0
100
200 km
80
60
40
20
0
count
0 100 200 300 400 500 600 700 800 900 1000 1100 1200
Geographical distance(km)
Cluster 3
0
100
200 km
150
100
50
0
count
0 100 200 300 400 500 600 700 800 900 1000 1100 1200
Geographical distance(km)
Cluster 4
0
100
200 km
400
300
200
100
0
count
0 100 200 300 400 500 600 700 800 900 1000 1100 1200
Geographical distance(km)

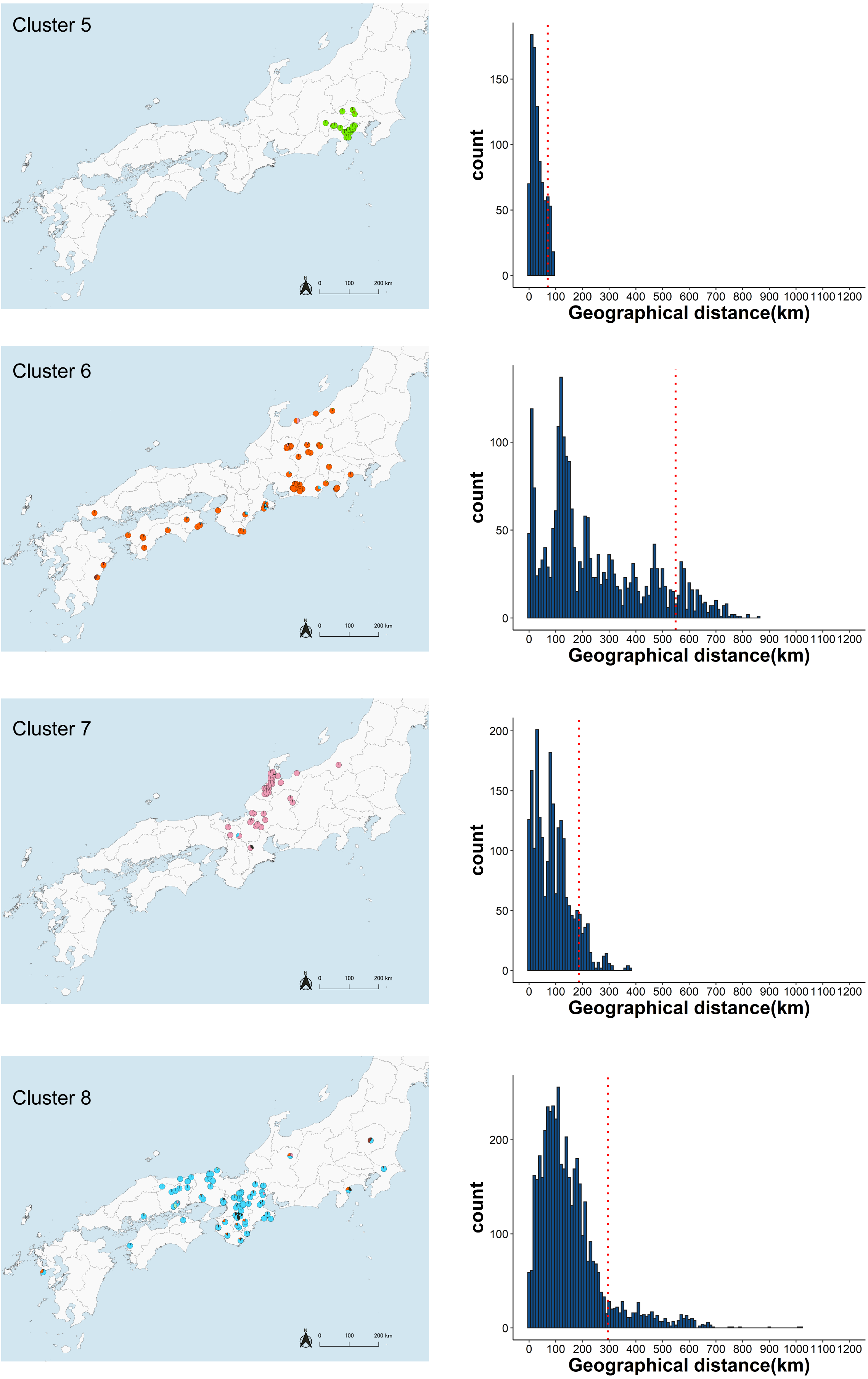

Cluster 5
Cluster 6
Cluster 7
Cluster 8
0 100 200 km
count
Geographical distance(km)
0 100 200 300 400 500 600 700 800 900 1000 1100 1200

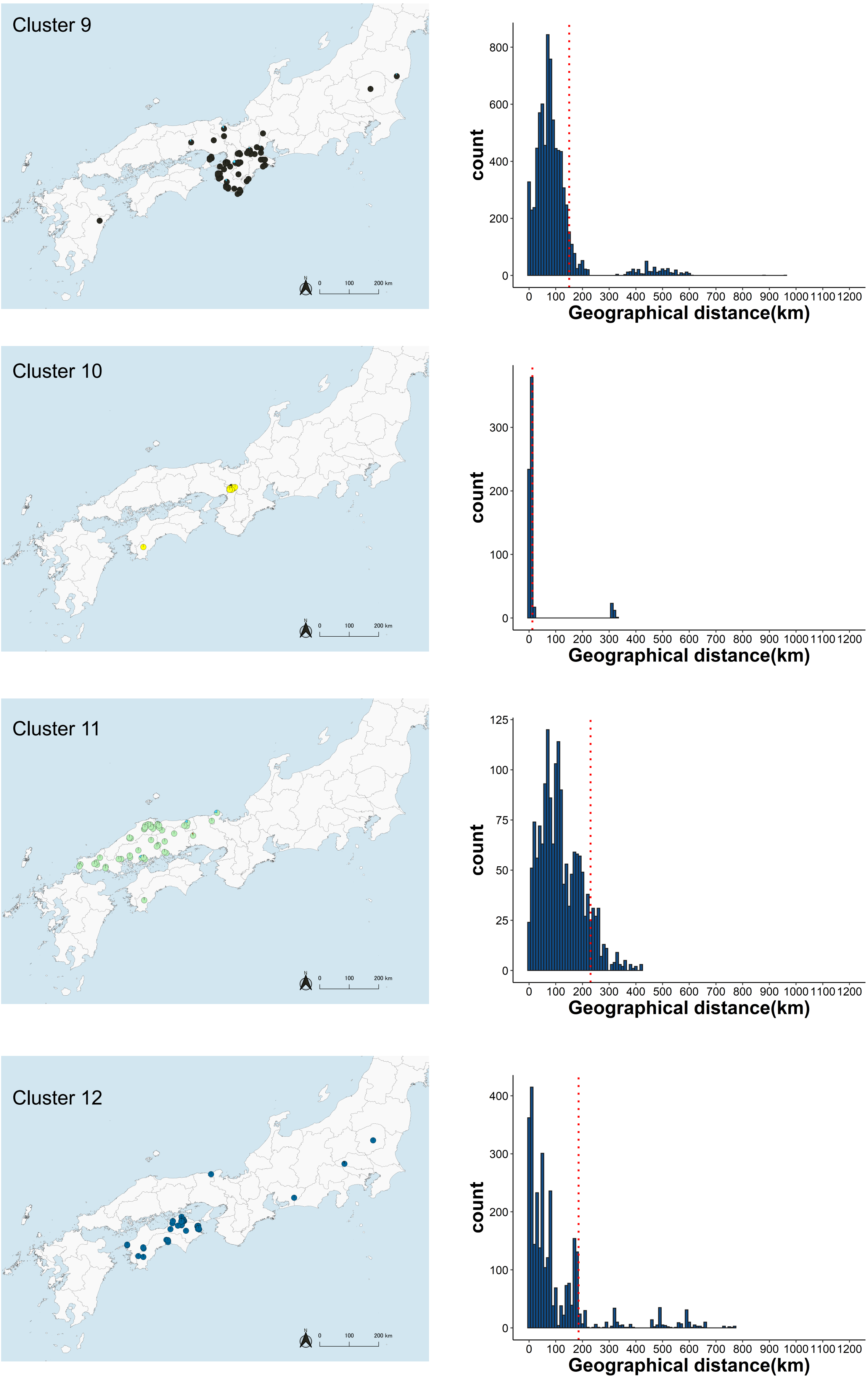
Cluster 9
N
0
100
200 km
count
800
600
400
200
0
0 100 200 300 400 500 600 700 800 900 1000 1100 1200
Geographical distance(km)
Cluster 10
N
0
100
200 km
count
300
200
100
0
0 100 200 300 400 500 600 700 800 900 1000 1100 1200
Geographical distance(km)
Cluster 11
N
0
100
200 km
count
125
100
75
50
25
0
0 100 200 300 400 500 600 700 800 900 1000 1100 1200
Geographical distance(km)
Cluster 12
N
0
100
200 km
count
400
300
200
100
0
0 100 200 300 400 500 600 700 800 900 1000 1100 1200
Geographical distance(km)

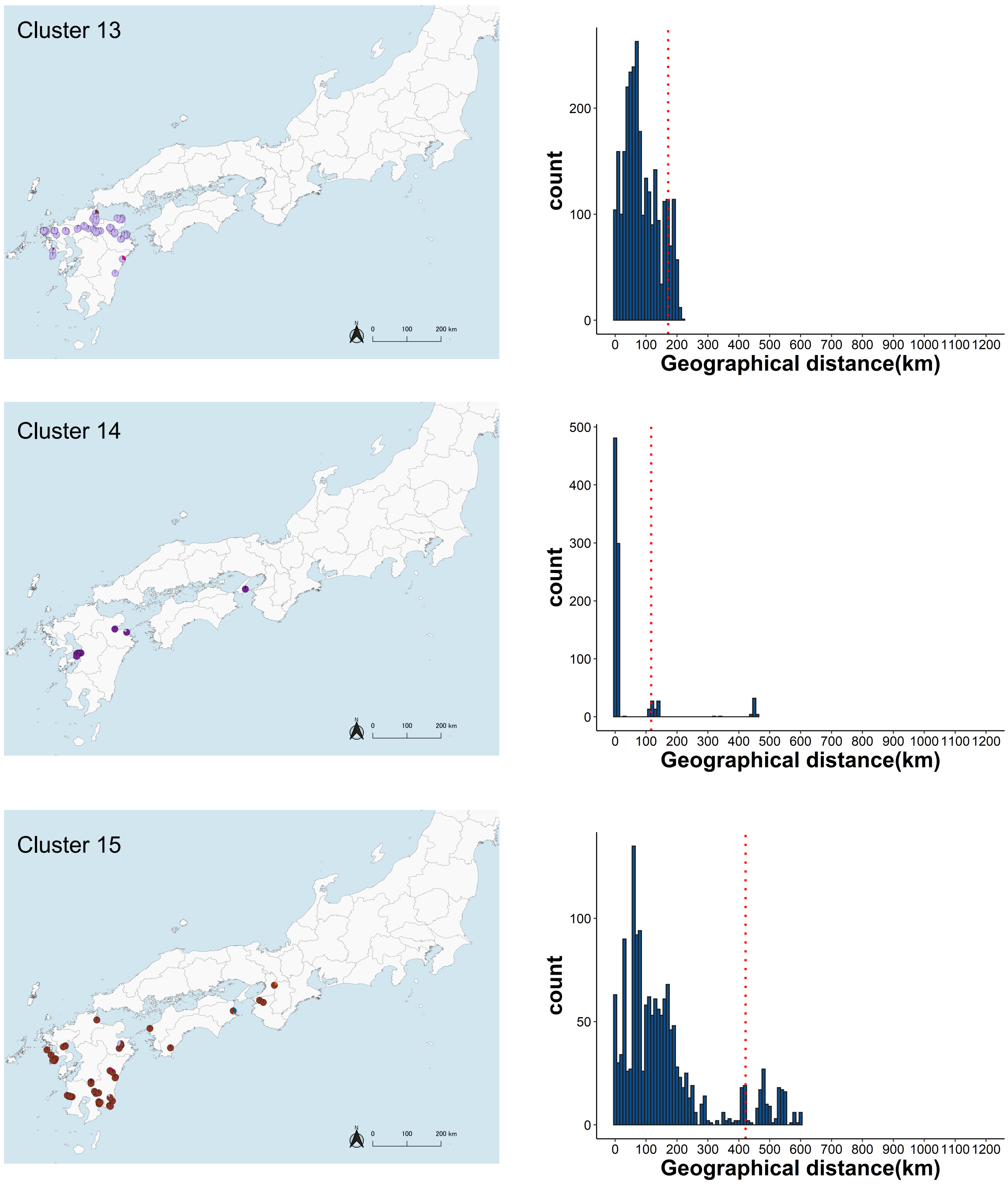

Supplementary Figure 2. Results of discriminant analysis of principal components (DAPC) analysis.

These figures show the distribution of individuals belonging to each cluster (left) and the pairwise geographic distance histogram (right). The red dotted line indicates the 90th percentile of distance within each cluster.

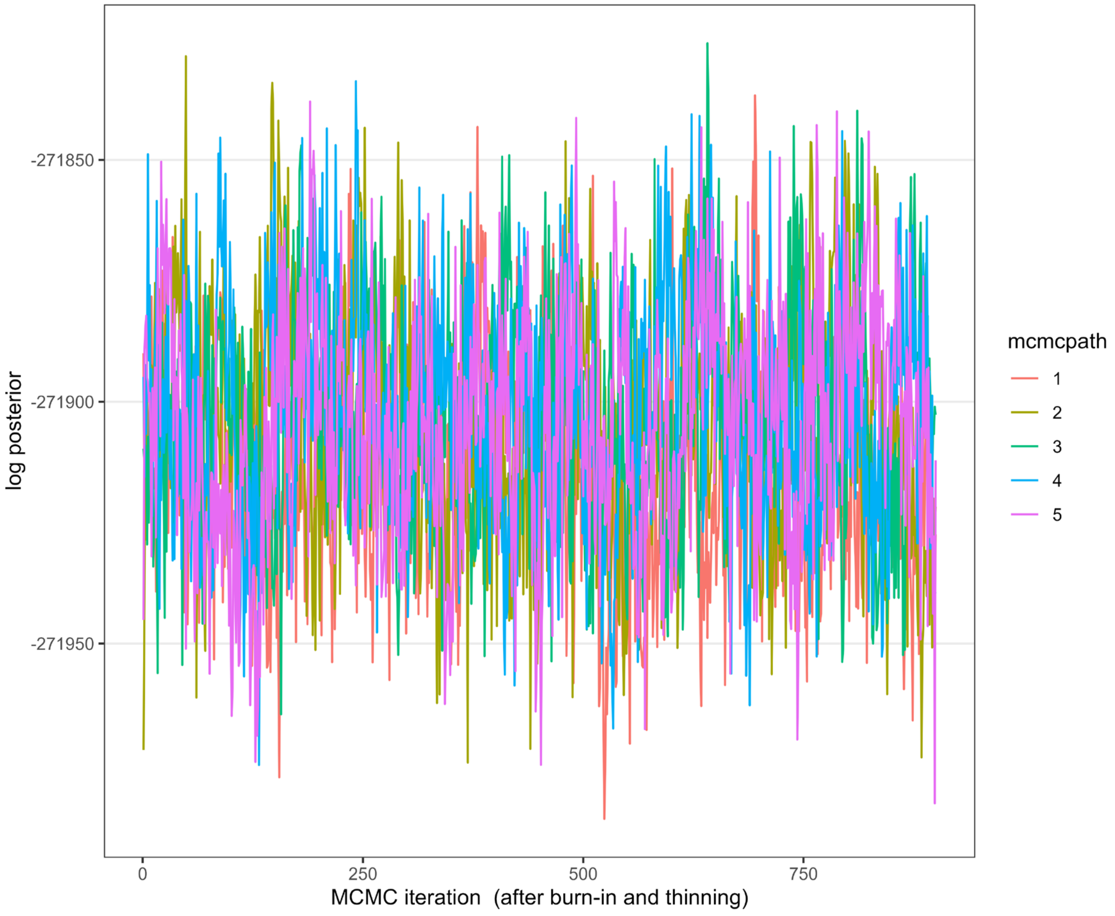

Supplementary Figure 3. EEMS output.

Posterior traces of five MCMC runs. We confirmed that all MCMC runs converged.

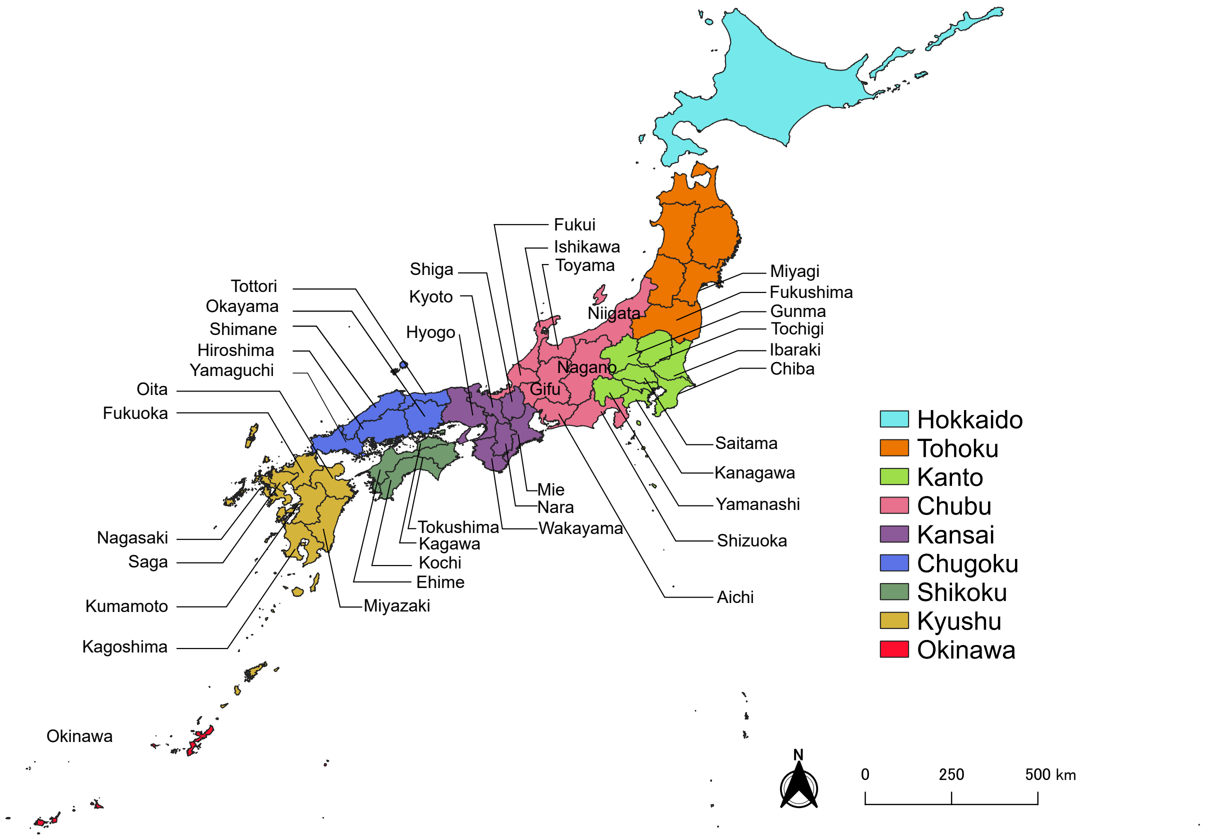

Supplementary Figure 4. Prefectures and regions of Japan used in this study.
